# Prognostic Value of Absolute Lymphocyte Count in Patients with Advanced Renal Cell Carcinoma Treated with Nivolumab plus Ipilimumab

**DOI:** 10.3390/jcm12062417

**Published:** 2023-03-21

**Authors:** Kosuke Ueda, Naoyuki Ogasawara, Naoki Ito, Satoshi Ohnishi, Hiroki Suekane, Hirofumi Kurose, Tasuku Hiroshige, Katsuaki Chikui, Keiichiro Uemura, Kiyoaki Nishihara, Makoto Nakiri, Shigetaka Suekane, Tsukasa Igawa

**Affiliations:** Department of Urology, Kurume University School of Medicine, Kurume 830-0011, Japan; ogasawara_naoyuki@med.kurume-u.ac.jp (N.O.); itou_naoki@kurume-u.ac.jp (N.I.); ohnishi_satoshi@med.kurume-u.ac.jp (S.O.); suekane_hiroki@med.kurume-u.ac.jp (H.S.); kurose_hirofumi@med.kurume-u.ac.jp (H.K.); hiroshige_tasuku@med.kurume-u.ac.jp (T.H.); chikui_katsuaki@med.kurume-u.ac.jp (K.C.); uemura_keiichirou@kurume-u.ac.jp (K.U.); nishihara_kiyoaki@kurume-u.ac.jp (K.N.); mnakiri@med.kurume-u.ac.jp (M.N.); suekane@med.kurume-u.ac.jp (S.S.); tigawa@med.kurume-u.ac.jp (T.I.)

**Keywords:** renal cell carcinoma, immune checkpoint inhibitor, nivolumab plus ipilimumab, lymphocyte count

## Abstract

Nivolumab and ipilimumab (NIVO + IPI) is standard therapy for patients with advanced renal cell carcinoma (RCC). Absolute lymphocyte count (ALC) is a valuable prognostic factor in patients with various cancers treated with immune checkpoint inhibitors. Herein, we determined the prognostic value of pretreatment ALC in advanced RCC patients treated with NIVO + IPI as first-line therapy. Data from 46 advanced RCC patients treated with NIVO + IPI between September 2018 and August 2022 were retrospectively reviewed and analyzed. Median progression-free survival (PFS) and overall survival (OS) were significantly shorter in patients with low than high ALC (PFS: *p* = 0.0095; OS: *p* = 0.0182). Multivariate analysis suggested that prior nephrectomy [hazard ratio (HR) = 3.854, 95% confidence interval (CI) = 1.433–10.359, *p* = 0.0075] and pretreatment ALC (HR = 2.513, 95% CI = 1.119–5.648, *p* = 0.0257) were independent factors for PFS. Our new prognostic ALNx model based on ALC and prior nephrectomy suggested that the poor-risk group was a predictor of significantly worse PFS (*p* < 0.0001) and OS (*p* = 0.0016). Collectively, the developed ALNx model may be a novel predictor of response in advanced RCC patients treated with NIVO + IPI.

## 1. Introduction

Immune checkpoint inhibitors (ICIs) targeting programmed cell death-1 (PD-1), programmed cell death-ligand 1 (PD-L1), and cytotoxic T-lymphocyte-associated antigen 4 (CTLA-4) have revolutionized the prognosis of advanced renal cell carcinoma (RCC) [1,2,3]. The International Metastatic Renal Cell Carcinoma Database Consortium (IMDC) risk score based on the results of the CheckMate 214 trial recommends the combination of nivolumab and ipilimumab (NIVO + IPI) as standard therapy for patients with advanced RCC with intermediate and poor risk [4]. Despite its valuable clinical applications, the efficacy of NIVO + IPI for RCC varies widely across individual patients. Therefore, reliable predictors must be identified to improve the prognosis of patients with advanced RCC who receive NIVO + IPI as first-line therapy.

Immune-related adverse events (irAEs) after ICI administration are considered effective biomarkers that correlate with the efficacy of these agents [5,6,7,8]. In a previous study, we reported that skin immune-related adverse events may be effective biomarkers in patients with advanced RCC treated with NIVO + IPI [5]. However, the appearance of irAEs does not predict the efficacy of ICIs at the start of treatment. Therefore, biomarkers that can predict treatment efficacy prior to drug administration must be identified. Recently, biomarkers that reflect the effects of various ICIs, such as tumor cell PD-L1 expression, tumor mutational burden, neoantigen burden, polybromo-1 gene mutation, immune cell infiltration, and intestinal microbiota, have been investigated [9]. However, biomarkers for the efficacy of NIVO + IPI remain unexplored. Pretreatment absolute lymphocyte count (ALC) can be used to predict the therapeutic effect of PD-1 monotherapy in various cancers [10,11,12]. Lymphocytes reflect the host immune function, which determines the overall success of ICI treatment. However, the significance of ALC in NIVO + IPI combination therapy remains unelucidated.

Therefore, we performed a retrospective analysis to identify biomarker predictors for the effect of NIVO + IPI in advanced RCC and developed a new prognostic model for combination therapy.

## 2. Materials and Methods

### 2.1. Study Design and Patients

We retrospectively examined the clinical information collected from 46 advanced RCC patients treated with NIVO + IPI as first-line therapy at the Kurume University Hospital, Japan, between September 2018 and August 2022. Patients with systemic inflammatory diseases were excluded from the study. Patients were stratified according to the IMDC risk classification at the introduction of NIVO + IPI therapy. In the induction phase, NIVO + IPI was administered intravenously at a dose of 240 mg/body and 1 mg/kg, respectively, every three weeks for four doses. Each drug, consisting of four doses, was administered every three weeks. In the maintenance phase, NIVO monotherapy was administered at a dose of 240 mg/body every two weeks or 480 mg/body every four weeks. Dose intervals were changed according to the condition of the patient. Pretreatment assessments of clinical characteristics and blood data were performed immediately before initiation of NIVO + IPI. Progression-free survival (PFS) was calculated from the initiation of NIVO + IPI to disease progression or death due to any cause. Overall survival (OS) was measured from NIVO + IPI initiation to death from any cause.

### 2.2. Statistical Analysis

Kaplan–Meier survival analysis and log-rank test were performed to compare survival between and among the studied cohorts, respectively. Univariate and multivariate analyses using the Cox proportional-hazards model were performed to identify risk factors for PFS and OS based on the calculation of hazard ratios (HRs) with 95% confidence intervals (CIs). Median values of ALC, C-reactive protein (CRP) level, and neutrophil-to-lymphocyte ratio (NLR) were considered the cutoff values. All statistical analyses were performed using JMP version 16 (SAS Institute Inc., Cary, NC, USA), and differences were considered significant at *p* < 0.05.

### 2.3. Ethical Approval

This study was conducted in compliance with the Declaration of Helsinki. The protocol was independently reviewed and approved by the Ethics Review Committee of the Kurume University School of Medicine. Given the retrospective nature of our study, the patients were not solicited for informed consent. All patient data were anonymized and de-identified prior to analysis.

## 3. Results

### 3.1. Patient Characteristics

The clinicopathological characteristics of the 46 study participants are shown in Table 1. The median age of the patients was 66.5 years (range: 42–80 years), and the majority of patients were men (84.8%). Twenty-three patients (50.0%) were each classified into intermediate- and poor-risk categories at NIVO + IPI initiation based on the IMDC risk classification. The percentage of patients who underwent nephrectomy before NIVO + IPI was 37.0%. The majority of patients were diagnosed with advanced RCC with clear cell histology (80.4%). The median PFS and OS of all patients were 12.4 (95% CI = 6.0–27.1) and 26.9 (95% CI = 22.9—not reached) months, respectively. The objective response rate was 51.1%. The median pretreatment ALC was 1289 (range: 524–3230) in all patients. We compared the clinicopathological characteristics of patients with high and low ALC before NIVO + IPI initiation. Patients with high ALC had a higher body mass index (BMI) than those with low ALC. In addition, patients with a low ALC had significantly higher NLR and CRP levels (NLR, *p* < 0.0001; CRP level, *p* = 0.0081). There was no significant difference in the Charlson comorbidity score between patients with low and high ALC (*p* = 1.000).

### 3.2. Clinical Course According to IMDC Risk Classification and Pretreatment Peripheral Inflammatory Biomarkers

**Figure 1:**
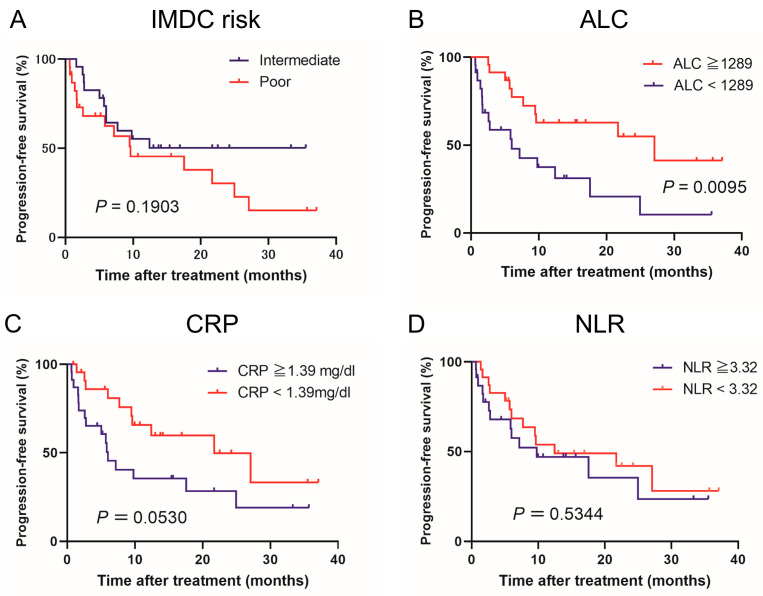
Kaplan–Meier curves comparing progression-free survival in patients with advanced renal cell carcinoma treated with nivolumab plus ipilimumab according to (**A**) International Metastatic Renal Cell Carcinoma Database Consortium (IMDC) risk classification, (**B**) absolute lymphocyte count (ALC), (**C**) C-reactive protein (CRP), and (**D**) neutrophil-lymphocyte ratio (NLR).

**Figure 2:**
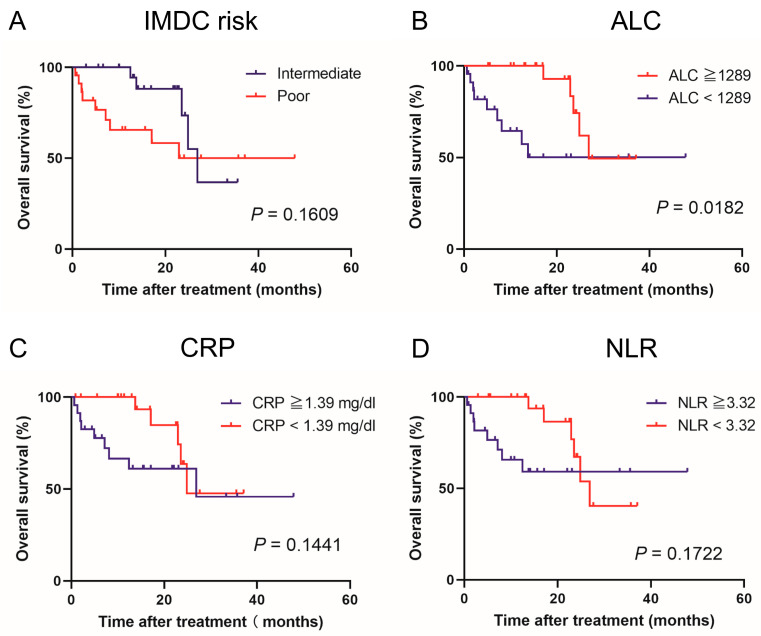
Kaplan–Meier curves comparing overall survival in patients with advanced renal cell carcinoma treated with nivolumab plus ipilimumab according to (**A**) International Metastatic Renal Cell Carcinoma Database Consortium (IMDC) risk classification, (**B**) absolute lymphocyte count (ALC), (**C**) C-reactive protein (CRP), and (**D**) neutrophil-lymphocyte ratio (NLR).

Figure 1 and Figure 2 show the estimated PFS and OS curves in patients with advanced renal cell carcinoma treated with NIVO + IPI according to the IMDC risk classifications and pretreatment peripheral inflammatory biomarkers, respectively. A low ALC was a predictor of significantly worse PFS (*p* = 0.0095) and OS (*p* = 0.0182) compared to a high ALC. However, there was no significant difference between the IMDC risk classifications. NLR findings were not associated with median PFS (*p* = 0.5344) or median OS (*p* = 0.1722). Regarding CRP levels, the PFS of patients in the high CRP group tended to be worse compared to patients in the low CRP group (*p* = 0.0530). However, no significant difference in PFS and OS was found between these two groups. No significant differences in PFS (*p* = 0.5344) and OS (*p* = 0.1722) were observed between the low and high NLR groups.

### 3.3. Univariate and Multivariate Analyses of Pretreatment Prognostic Factors

Univariate and multivariate analyses were performed using the Cox proportional hazards model to identify the pretreatment prognostic factors before NIVO + IPI initiation associated with PFS and OS (Table 2 and Table 3). Univariate analysis revealed that prior nephrectomy (HR = 4.162, 95% CI = 1.554–11.148, *p* = 0.0046) and pretreatment ALC (HR = 2.762, 95% CI = 1.241–6.149, *p* = 0.0128) were significant factors affecting PFS. Multivariate analyses suggested that prior nephrectomy (HR = 3.854, 95% CI = 1.433–10.359, *p* = 0.0075) and pretreatment ALC (HR = 2.513, 95% CI = 1.119–5.648, *p* = 0.0257) had independent prognostic effects on PFS. In univariate analysis related to OS, sex (HR = 4.093, 95% CI = 1.211–13.835, *p* = 0.0233) and pretreatment ALC (HR = 3.564, 95% CI = 1.166–10.896, *p* = 0.0258) were significant factors that affected OS. Multivariate analyses also suggested that sex (HR = 3.659, 95% CI = 1.055–12.687, *p* = 0.0409) and pretreatment ALC (HR = 3.367, 95% CI = 1.075–10.541, *p* = 0.0371) had independent prognostic effects on OS.

### 3.4. Prognostic Model Using Prior Nephrectomy and ALC (ALNx Model)

**Figure 3:**
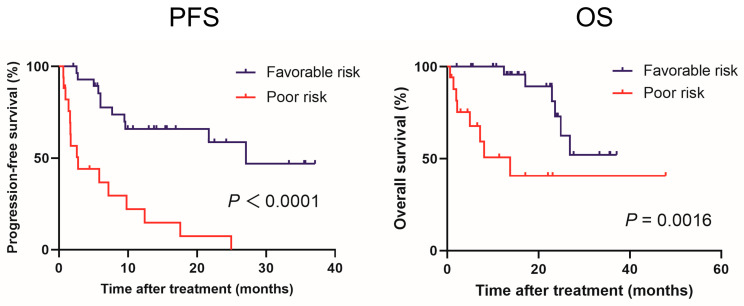
Kaplan–Meier curves comparing progression-free survival and overall survival in patients with advanced renal cell carcinoma treated with nivolumab plus ipilimumab according to the ALNx model.

Based on the results of multivariate analyses for PFS, a prognostic model, called the ALNx model, was developed to predict the effect of NIVO + IPI by defining prior nephrectomy and pretreatment ALC as risk factors. In this model, patients were stratified into two groups according to the presence or absence of the aforementioned independent risk factors: the favorable-risk group (patients with 0 or 1 risk factor) and the poor-risk group (those with 2 risk factors) (Figure 3). The poor-risk group was a predictor of significantly worse PFS (*p* < 0.0001) and OS (*p* = 0.0016) compared with the favorable-risk group. No difference was observed in PFS and OS between patients with 0 and 1 risk factors (Supplementary Materials Figure S1).

## 4. Discussion

The combination of NIVO + IPI provides a favorable treatment response and prolongs survival. However, the biomarker for predicting treatment response remains unclear. In an exploratory analysis of the CheckMate 214 trial, Motzer et al. reported the importance of the inflammatory status of the tumor microenvironment in identifying predictive biomarkers of response and survival with NIVO + IPI combination therapy in RCC patients [13]. Systemic inflammatory factors, including NLR, platelet-to-lymphocyte ratio (PLR), CRP level, and systemic immune inflammation (SII), have the potential to predict survival outcomes in patients receiving NIVO + IPI for metastatic RCC [14,15]. Hematological parameters, such as NLR, PLR, and SII, reflect the balance between the inflammation and immune response. Iinuma et al. reported that pretreatment NLR was a prognostic factor for survival in RCC patients treated with NIVO +IPI [15]. In contrast, pretreatment NLR is not a significant predictor of response to NIVO + IPI therapy [16,17]. In our study, pretreatment NLR was not significantly associated with either PFS or OS; therefore, the utility of NLR in NIVO + IPI therapy remains controversial and must be further elucidated.

Serum CRP is a known biomarker of systemic inflammatory reactions. Systemic inflammation measured by CRP plays a crucial role in the prognosis of metastatic RCC treated with molecular-targeted therapies [18,19,20]. Ishihara et al. demonstrated that pretreatment CRP level was a predictive factor for OS but not for PFS in patients treated with nivolumab therapy for metastatic RCC [21]. With NIVO + IPI as the primary treatment regimen, Yano et al. reported that the OS for patients with high CRP levels was significantly shorter than for those with low CRP levels [14]. However, no significant correlation was observed between CRP level and PFS for NIVO + IPI in their study. These results suggest that pretreatment CRP levels reflect the prognosis of RCC in patients treated with antitumor drugs but may not reflect the effectiveness of ICIs.

Lymphocytes are the most critical actors in the adaptive immune system during the anticancer immune response. Furthermore, ALC represents the state of immune function in the patient, and lymphopenia may be associated with a poor prognosis in patients receiving ICIs. Low ALC before ICI initiation is a predictor of poor response in many cancers, including lung cancer, esophageal cancer, head and neck cancer, and melanoma [11,12,22,23,24]. We previously reported that ALC was an independent biomarker of therapeutic response in patients with metastatic RCC treated with nivolumab monotherapy [10].

Currently, the IMDC risk classification is widely used to categorize risk in metastatic RCC [25]. However, this risk classification reflects the prognosis in the molecular targeted therapy era, and there is controversy as to whether the outcomes can be used as predictors of the efficacy of ICIs, especially NIVO + IPI. Escudier et al. showed that the objective response rate does not differ significantly by the number of IMDC risk factors in patients with advanced RCC treated with NIVO + IPI [26]. We analyzed the prognosis of patients treated with NIVO + IPI as first-line therapy using the factors listed as IMDC risk factors in order to develop a new risk model. In this retrospective study, low ALC correlated with a worse response to NIVO + IPI. Moreover, we observed that pretreatment ALC and prior nephrectomy were independent prognostic factors for PFS.

Cytoreductive nephrectomy (CN) is not recommended for patients with poor prognostic features based on several retrospective studies as well as the results of the CARMENA and SURTIME trials [27,28]. However, these studies validated the efficacy of CN in the era of molecular targeted therapy. Tanaka et al. demonstrated that response to NIVO + IPI therapy was not affected by prior nephrectomy [29]. Contrarily, Kato et al. reported that NIVO + IPI was associated with a better outcome in patients who had undergone prior nephrectomy for synchronous metastatic RCC [30]. Albiges et al. reported that in the CheckMate 214 trial, 35% of patients treated with NIVO + IPI who had not undergone nephrectomy achieved a 30% or more reduction in target kidney lesions [31]. As there is no definite evidence regarding the efficacy of NIVO + IPI in patients who have not undergone nephrectomy, further studies are needed to validate the importance of nephrectomy prior to NIVO + IPI therapy.

To develop a model for predicting the efficacy of the NIVO + IPI combination, we compared the survival curves generated using the ALNx model. The median PFS stratified by our risk classification was 27.1 and 2.8 months for the favorable and poor risk groups, respectively. The median OS stratified by our risk classification was 13.8 months in the poor-risk group compared to “not reached” in the favorable-risk group. Collectively, these results suggest that our ALNx model may be valuable for predicting the treatment efficacy of NIVO + IPI combination therapy for prognostic stratification. Unlike the evaluation of tumor-infiltrating lymphocytes (TILs) and PD-L1 expression, this model, which includes simple and easy-to-use biomarkers, can be implemented without a pathologist.

Our present study has several limitations. First, this was a retrospective study comprising a small number of patients from a single institution. Univariate and multivariate analyses of OS indicated that survival after initiation of NIVO + IPI was significantly better in men than in women. This finding contrasts with the results of a previous study that found a higher survival rate in women with RCC. This difference may be attributed to the small number of patients and the selection bias in our study. The presence of concurrent inflammatory states or the use of immunomodulators, which could have affected the inflammatory markers, has not been validated. Previously reported biomarkers, such as PD-L1 expression and levels of TILs in renal tumors, were not examined in this study. Moreover, a few reports have demonstrated that peripheral blood ALC and TILs are correlated [32,33]. A recent study showed that myeloid inflammation plays a crucial role in the pathogenesis of metastatic clear cell RCC [34]. However, this correlation was not examined in this study. Therefore, further validation in larger and more diverse populations is required to comprehensively elucidate whether ALC before NIVO + IPI combination therapy can predict PFS and OS in advanced RCC.

## 5. Conclusions

In conclusion, our study indicated that peripheral ALC before the initiation of NIVO + IPI was a predictor of poor response in patients with advanced RCC. Additionally, the ALNx model, when using ALC and prior nephrectomy, may be a novel predictor of response in patients with advanced RCC treated with NIVO + IPI. We believe that this study provides valuable insights into the prognosis of patients with advanced RCC undergoing NIVO + IPI therapy.

## Figures and Tables

**Table 1:** Clinical and pathological features by absolute lymphocyte count in patients with advanced renal cell carcinoma treated with nivolumab plus ipilimumab.

Feature		Total (*n* = 46)	ALC ≥ 1289 (*n* = 23)	ALC < 1289 (*n* = 23)	*p*
Age, years, range		66.5 (42–80)	67 (42–80)	66.5 (48–80)	0.5974
Sex, *n* (%)	Male	39 (84.8)	21 (91.3)	18 (78.3)	0.2118
	Female	7 (15.2)	2 (8.7)	5 (21.7)	
BMI (kg/m^2^), median		22.7	24.6	21.4	0.0098
Prior nephrectomy, *n* (%)	Yes	17 (37.0)	11 (47.8)	6 (26.1)	0.1246
	No	29 (63.0)	12 (52.2)	17 (73.9)	
Performance status, *n* (%)	0, 1	39 (84.8)	19 (82.6)	20 (87.0)	0.6810
	≥2	7 (15.2)	4 (17.4)	3 (13.0)	
Histological subtype, *n* (%)	CCRCC	37 (80.4)	20 (87.0)	17 (73.9)	0.4557
	Non-CCRCC	7 (15.2)	2 (8.7)	5 (21.7)	
	Unknown	2 (4.3)	1 (4.3)	1 (4.3)	
IMDC risk classification, *n* (%)	Intermediate	23 (50.0)	14 (60.9)	9 (39.1)	0.1388
	Poor	23 (50.0)	9 (39.1)	14 (60.9)	
Liver metastasis, *n* (%)	No	42 (91.3)	21 (91.3)	21 (91.3)	1.0000
	Yes	4 (8.7)	2 (8.7)	2 (8.7)	
Bone metastasis, *n* (%)	No	33 (71.7)	17 (73.9)	16 (69.6)	0.7432
	Yes	13 (28.3)	6 (26.1)	7 (30.4)	
Charlson comorbidity score, *n* (%)	≤2	44 (95.7)	22 (95.7)	22 (95.7)	1.0000
	≥3	2 (4.3)	1 (4.3)	1 (4.3)	
Monocyte count, median (range)		399.5 (128.7–620.0)	396.5 (255.0–600.6)	402.1 (128.7–620.0)	0.8519
NLR, median (range)		3.32 (1.03–11.51)	2.30 (1.03–4.16)	4.99 (2.65–11.51)	<0.0001
CRP, mg/dL, median (range)		1.39 (0.08–18.58)	0.62 (0.08–7.36)	3.27 (0.32–18.58)	0.0081

ALC, absolute lymphocyte count; BMI, body mass index; CCRCC, clear cell renal cell carcinoma; IMDC, International Metastatic Renal Cell Carcinoma Database Consortium; NLR, neutrophil-lymphocyte ratio; CRP, C-reactive protein.

**Table 2:** Univariate and multivariate analyses of progression-free survival in patients with advanced renal cell carcinoma treated with nivolumab plus ipilimumab.

	Progression-Free Survival (*n* = 46)			
	Univariate Analysis		Multivariate Analysis	
Variable	HR	*p*-Value	HR	*p*-Value
Age (≥70 years)	0.815 (0.342–1.940)	0.6434		
Sex (female)	2.016 (0.748–5.436)	0.1657		
Prior nephrectomy (no)	4.162 (1.554–11.148)	0.0046	3.854 (1.433–10.359)	0.0075
Performance status (≥2)	1.393 (0.474–4.090)	0.5467		
IMDC risk classification (poor)	1.678 (0.766–3.675)	0.1957		
Liver metastasis (yes)	1.175 (0.276–5.012)	0.8271		
Bone metastasis (yes)	1.546 (0.686–3.484)	0.2934		
CRP (mg/dL) (≥1.39)	2.149 (0.971–4.756)	0.0591		
ALC (<1289)	2.762 (1.241–6.149)	0.0128	2.513 (1.119–5.648)	0.0257
Monocyte count (≥399.5)	1.419 (0.653–3.084)	0.3768		
Anemia (yes)	4.971 (0.672–36.785)	0.1164		
Calcium (upper limit over)	1.712 (0.753–3.888)	0.1993		
Neutrophil count (upper limit over)	1.970 (0.671–5.783)	0.2169		
Platelet count (upper limit over)	1.349 (0.507–3.587)	0.5486		

IMDC, International Metastatic Renal Cell Carcinoma Database Consortium; CRP, C-reactive protein; ALC, absolute lymphocyte count; HR, hazard ratio.

**Table 3:** Univariate and multivariate analyses of overall survival in patients with advanced renal cell carcinoma treated with nivolumab plus ipilimumab.

	Overall Survival (*n* = 46)			
	Univariate Analysis		Multivariate Analysis	
Variable	HR	*p*-Value	HR	*p*-Value
Age (≥70 years)	0.385 (0.086–1.723)	0.2118		
Sex (female)	4.093 (1.211–13.835)	0.0233	3.659 (1.055–12.687)	0.0409
Prior nephrectomy (no)	3.078 (0.854–11.089)	0.0856		
Performance status (≥2)	3.814 (1.138–12.790)	0.0301		
IMDC risk classification (poor)	2.156 (0.718–6.476)	0.1709		
Liver metastasis (yes)	0.675 (0.087–5.210)	0.7059		
Bone metastasis (yes)	1.408 (0.468–4.238)	0.5424		
CRP (mg/dL) (≥1.39)	2.455 (0.834–7.225)	0.1029		
ALC (<1289)	3.564 (1.166–10.896)	0.0258	3.367 (1.075–10.541)	0.0371
Monocyte count (≥399.5)	2.789 (0.868–8.962)	0.0851		
Anemia (yes)	1.955 (0.255–14.980)	0.5188		
Calcium (upper limit over)	1.116 (0.342–3.639)	0.8551		
Neutrophil count (upper limit over)	3.013 (0.831–10.930)	0.0934		
Platelet count (upper limit over)	2.995 (0.924–9.706)	0.0675		

IMDC, International Metastatic Renal Cell Carcinoma Database Consortium; CRP, C-reactive protein; ALC, absolute lymphocyte count; HR, hazard ratio.

## Data Availability

Not applicable.

## Supplementary Materials

The following supporting information can be downloaded at: https://www.mdpi.com/article/10.3390/jcm12062417/s1, Figure S1: Kaplan–Meier estimates of progression-free survival and overall survival in patients with advanced renal cell carcinoma treated with nivolumab plus ipilimumab according to risk classification by prior nephrectomy and absolute lymphocyte count.
