# Supplementary material for: Prognostic Value of Absolute Lymphocyte Count in Patients with Advanced Renal Cell Carcinoma Treated with Nivolumab plus Ipilimumab
Source: J Clin Med. 2023 Mar 21;12(6):2417. doi: 10.3390/jcm12062417 (PMC10053370; doi:10.3390/jcm12062417)
Supplement: Supplementary file 1 [file jcm-12-02417-s001.zip › jcm-2236556-supplementary.pdf]

Journal of
Clinical Medicine

Article

Prognostic Value of Absolute Lymphocyte Count in Patients with Advanced Renal Cell Carcinoma Treated with Nivolumab Plus Ipilimumab

Kosuke Ueda *, Naoyuki Ogasawara, Naoki Ito, Satoshi Ohnishi, Hiroki Suekane, Hirofumi Kurose, Tasuku Hiroshige, Katsuaki Chikui, Keiichiro Uemura, Kiyoaki Nishihara, Makoto Nakiri, Shigetaka Suekane and Tsukasa Igawa

Department of Urology, Kurume University School of Medicine, Kurume, Japan
* Correspondence: ueda_kousuke@med.kurume-u.ac.jp; Tel.: +81-942-31-7572

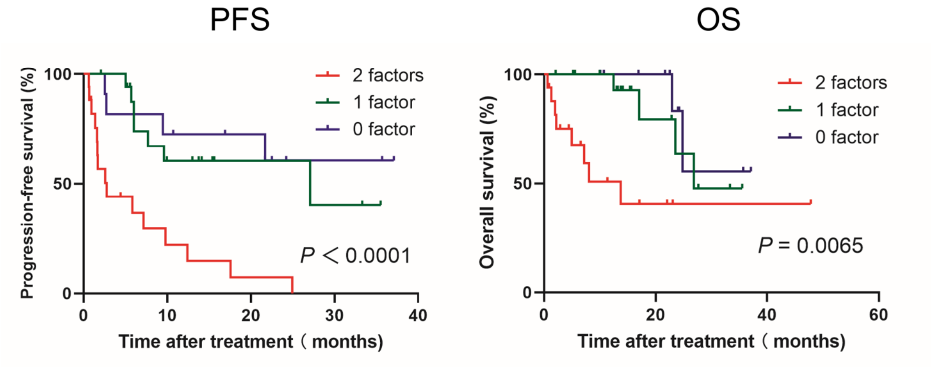

Figure S1. Kaplan–Meier curves comparing progression-free survival and overall survival in patients with advanced renal cell carcinoma treated with nivolumab plus ipilimumab according to risk classification by prior nephrectomy and absolute lymphocyte count.

Citation: Ueda, K.; Ogasawara, N.; Ito, N.; Ohnishi, S.; Suekane, H.; Kurose, H.; Hiroshige, T.; Chikui, K.; Uemura, K.; Nishihara, K.; et al. Prognostic Value of Absolute Lymphocyte Count in Patients with Advanced Renal Cell Carcinoma Treated with Nivolumab Plus Ipilimumab. *J. Clin. Med.* **2023**, *12*, 2417. https://doi.org/10.3390/jcm12062417

Academic Editor: Cristian Fiori

Received: 7 February 2023
Revised: 13 March 2023
Accepted: 20 March 2023
Published: 21 March 2023

Copyright: © 2023 by the authors. Submitted for possible open access publication under the terms and conditions of the Creative Commons Attribution (CC BY) license (https://creativecommons.org/licenses/by/4.0/).
